# Prevention of delirium with agitation by yokukansan in older adults after cancer surgery

**DOI:** 10.1093/jjco/hyac123

**Published:** 2022-07-30

**Authors:** Ryoichi Sadahiro, Saho Wada, Yutaka J Matsuoka, Yosuke Uchitomi, Takuhiro Yamaguchi, Tetsufumi Sato, Minoru Esaki, Seiichi Yoshimoto, Hiroyuki Daiko, Yukihide Kanemitsu, Akira Kawai, Tomoyasu Kato, Hiroyuki Fujimoto, Yasuhito Uezono, Ken Shimizu, Hiromichi Matsuoka

**Affiliations:** Department of Psycho-Oncology, National Cancer Center Japan, Tokyo, Japan; Department of Psycho-Oncology, National Cancer Center Japan, Tokyo, Japan; Department of Neuropsychiatry, Nippon Medical School, Tama Nagayama Hospital, Tokyo, Japan; Former Division Chief of Health Care Research, Center for Public Health Sciences, National Cancer Center Japan, Tokyo, Japan; Department of Psycho-Oncology, National Cancer Center Japan, Tokyo, Japan; Group for Supportive Care and Survivorship Research, Institute for Cancer Control, National Cancer Center Japan, Tokyo, Japan; Division of Biostatistics, Tohoku University Graduate School of Medicine, Miyagi, Japan; Department of Anesthesia and Intensive Care, National Cancer Center Japan, Tokyo, Japan; Department of Hepatobiliary and Pancreatic Surgery, National Cancer Center Japan, Tokyo, Japan; Department of Head and Neck Surgery, National Cancer Center Japan, Tokyo, Japan; Department of Esophageal Surgery, National Cancer Center Japan, Tokyo, Japan; Department of Colorectal Surgery, National Cancer Center Japan, Tokyo, Japan; Department of Musculoskeletal Oncology and Rehabilitation, National Cancer Center Japan, Tokyo, Japan; Department of Gynecology, National Cancer Center Japan, Tokyo, Japan; Department of Urology, National Cancer Center Japan, Tokyo, Japan; Department of Pain Control Research, Jikei University School of Medicine, Tokyo, Japan; Department of Psycho-Oncology, Cancer Institute Hospital of Japanese Foundation for Cancer Research, Tokyo, Japan; Department of Psycho-Oncology, National Cancer Center Japan, Tokyo, Japan

**Keywords:** clinical trial, surgical oncology, psycho-oncology, gerontology

## Abstract

**Objective:**

Preventing postoperative delirium with agitation is vital in the older population. We examined the preventive effect of yokukansan on postoperative delirium with agitation in older adult patients undergoing highly invasive cancer resection.

**Methods:**

We performed a secondary per-protocol analysis of 149 patients’ data from a previous clinical trial. Patients underwent scheduled yokukansan or placebo intervention 4–8 days presurgery and delirium assessment postoperatively. Delirium with agitation in patients aged ≥75 years was assessed using the Diagnostic and Statistical Manual of Mental Disorders, Fifth Edition, and the Japanese version of the Delirium Rating Scale-Revised-98. We assessed odds ratios for yokukansan (TJ-54) compared with placebo for the manifestation of postoperative delirium with agitation across patients of all ages (*n* = 149) and those aged ≥65 years (*n* = 82) and ≥ 75 years (*n* = 21) using logistic regression.

**Results:**

Delirium with agitation manifested in 3/14 and 5/7 patients in the TJ-54 and placebo groups, respectively, among those aged ≥75 years. The odds ratio for yokukansan vs. placebo was 0.11 (95% confidence interval: 0.01–0.87). An age and TJ-54 interaction effect was detected in patients with delirium with agitation. No intergroup differences were observed in patients aged ≥65 years or across all ages for delirium with agitation.

**Conclusions:**

This is the first study investigating the preventive effect of yokukansan on postoperative delirium with agitation in older adults. Yokukansan may alleviate workforce burdens in older adults caused by postoperative delirium with agitation following highly invasive cancer resection.

## Teaser

This secondary per-protocol analysis demonstrated that yokukansan is preventive against postoperative delirium with agitation in patients aged ≥75 years undergoing invasive cancer resection, and therefore could alleviate workforce burdens.

## Introduction

In older populations, postoperative delirium has substantial economic and societal impacts and is considered an indicator of quality of care (1,2). Postoperative delirium results not only in short-term adverse events, such as extended hospital stays and increased mortality (3–5), but also long-term prognoses, which include increased mortality, dementia and institutionalization (6). Although clinical guidelines recommend a multicomponent non-pharmacological program, pharmacologic treatment is not recommended for the prevention of postoperative delirium (7), despite the apparent efficacy of several agents (8). Reasons for this include dose-dependent sedation, extrapyramidal symptoms and QT prolongation syndrome induced by the use of antipsychotics in patients with postoperative delirium (9), which can lead to increased mortality in older adults and patients with dementia (10,11). Therefore, developing drug therapies to prevent postoperative delirium with few side effects is an important clinical challenge.

We previously conducted a randomized, double-blind, placebo-controlled trial to investigate whether yokukansan, a Japanese herbal medicine, reduces the incidence of postoperative delirium in patients undergoing invasive cancer resection (12). Although we focused on yokukansan as a preventive drug for postoperative delirium in accordance with reports of its effectiveness in the treatment of behavioural and psychological symptoms of dementia (BPSD) that resemble delirium (13,14), yokukansan did not reduce the incidence of postoperative delirium. There are several possible explanations for this result.

First, differences in study setting between our study and previous studies may have influenced the negative result. In particular, we considered the age of participants an important factor that influences the efficacy of yokukansan. Older age is considered a major independent risk factor for delirium, with an increase in delirium risk with age from 3% in those aged under 65 years, to 14% in those aged 65 to 74 years, and to 36% in patients aged 75 years and older. Moreover, aging is associated with several age-related cerebral reserves, which include changes in the proportion of stress-regulating neurotransmitters, brain blood flow decline and neuronal loss (15). Previous studies on BPSD (16–19) targeted participants aged 55 years and over and examined efficacy in older adult populations (mean ages were 78 years or older); however, our study targeted patients aged 20 years and over, and approximately 25% of participants were aged under 55 years (mean age 63 years) (12). These age differences, and thus differences in cerebral reserves, may have influenced the outcomes.

Second, yokukansan may be effective for only certain delirium symptoms. In our study, yokukansan tended to reduce the severity of delirium, as assessed using the Japanese version of the Delirium Rating Scale-Revised-98 (DRS-R-98), which evaluates a broad range of delirium symptoms, such as orientation, attention, hallucination and agitation. Of these various symptoms, our study focused on agitation because previous studies investigating the effects of yokukansan on diseases other than delirium showed that yokukansan was particularly effective for treating agitation. For example, Furukawa et al. reported that yokukansan improved agitation/aggression in patients with Alzheimer’s disease (16), and Miyaoka et al. showed that yokukansan improved excitement/hostility symptoms in patients with schizophrenia (20).

Therefore, according to the above considerations, we hypothesized that yokukansan would (i) reduce postoperative delirium in older adults and (ii) prevent postoperative delirium with agitation. To clarify these two hypotheses, we performed a secondary analysis of a double-blind randomized controlled trial to determine whether yokukansan prevents postoperative delirium with agitation in patients aged 75 years or older undergoing highly invasive cancer resection.

## Methods

### Study design and oversight

This was a secondary analysis of a single-centre, randomized, double-blind, placebo-controlled trial called the ProD study. The study design and results of the ProD study are reported elsewhere (12,21). Briefly, 195 participants undergoing invasive cancer resection, with a scheduled surgical time of 6 hours or longer, were recruited at the National Cancer Center Hospital in Japan and randomized to receive 7.5 g daily of yokukansan as Tsumura Yokukansan Extract Granules (TJ-54, Tsumura & Co., Tokyo, Japan) orally or placebo. The study was stopped by an interim report with a mean preoperative anxiety score of 5.0 as assessed using the Hospital Anxiety and Depression Scale-Anxiety (22) and a 42% incidence of postoperative delirium diagnosed according to the Diagnostic and Statistical Manual of Mental Disorders, Fifth Edition (DSM-5) (23) during the 5 days following surgery in the yokukansan group. Statistical analysis of the full dataset was performed in 160 participants and showed that yokukansan was not substantially effective in treating preoperative anxiety or preventing postoperative delirium. The trial was registered in the UMIN Clinical Trials Registry (https://upload.umin.ac.jp/cgi-open-bin/ctr/ctr_view.cgi?recptno=R0000314888; UMIN000027561) and approved by the Institutional Review Board and the Ethics Committee of the National Cancer Center in Japan before starting enrolment. We received written informed consent from all patients who participated in this study.

### Participants

Among the 195 participants recruited into the ProD study, this secondary analysis was a per-protocol analysis of 149 participants who completed the intervention protocol as scheduled, 4–8 days before surgery, and underwent delirium assessment postoperatively.

### Outcomes

The primary outcome was the incidence of postoperative delirium with agitation in patients aged 75 years or older. Secondary outcomes were the development of any delirium across all age groups and in the 65 years or older group, and time to onset of postoperative delirium with agitation and any delirium. Participants were assessed for delirium using the DSM-5 and DRS-R-98 (24,25) during the 5 days following surgery. The kappa value for inter-rater reliability for the DSM-5 was 0.85. ‘Delirium with agitation’ according to the DSM-5 and a score of 1 or higher on the agitation subscale of the DRS-R-98 were defined as a positive diagnosis, as reported previously (26). The following characteristics were also assessed: age, sex, body mass index (BMI), cognitive function (as assessed using the Mini-Mental State Examination [MMSE]), Charlson’s Comorbidity Index (CCI), primary cancer location, educational duration, American Society of Anaesthesiologists (ASA) class, alcohol use and performance status.

### Statistical analysis

Efficacy of yokukansan for the prevention of postoperative delirium with agitation was assessed across all ages and also in stratified groups of 65 years or older and 75 years or older, the latter of whom have a high delirium risk (15). Patient characteristics were compared between groups using *t*-tests and Fisher’s exact tests. Logistic regression was performed to estimate the odds ratio for yokukansan for the prevention of postoperative delirium with a 95% confidence interval. The interaction effect between age and yokukansan was tested using an interaction term in the logistic regression, with centralized dummy variables of age (75 years or older = 1, younger than 75 years = 0) and intervention (yokukansan = 1, placebo = 0). Time to delirium onset was also analyzed to determine the effect of yokukansan using the log-rank test with the Kaplan–Meier method. A *P* value of <0.05 was considered significant. Analyses were conducted using R version 4.1.0 (https://www.r-project.org). We did not impute any missing data.

## Results

The characteristics of patients aged 75 years or older are described in Table 1. Those of all ages and the 65 years or older group are listed in Supplementary Tables S1 and S2. In the 75 years or older group, education duration was shorter in the TJ-54 group (Table 1). BMI was higher in the TJ-54 group across all ages and in the 65 years or older group (Supplementary Tables S1 and S2). For the primary outcome, delirium with agitation was detected in three patients (28.6%) in the TJ-54 group and five patients (71.4%) in the placebo group among those aged 75 years or older (Table 2). Any delirium was observed in seven and five participants (50.0% and 71.4%) in the TJ-54 and placebo groups, respectively, among those aged 75 years or older (Table 2). The logistic regression yielded odds ratios for yokukansan of 0.11 (95% confidence interval: 0.01–0.87) for delirium with agitation and 0.40 (95% confidence interval: 0.06–2.80) for any delirium (Table 2). There was a significant interaction effect between age and TJ-54 for delirium with agitation (odds ratio: 0.07, 95% confidence interval: 0.01–0.80; *P* = 0.03), which indicated an odds ratio for yokukansan for postoperative delirium with agitation of 0.58 among those aged 75 years or older compared with those younger than 75 years. There was no interaction between age and TJ-54 for any delirium (odds ratio: 0.33, 95% confidence interval: 0.04–2.66; *P* = 0.30).

**Table 1:** Characteristics of participants aged 75 years or older

			Intervention group		Placebo group		*P* value
Characteristic			(*n* = 14)		(*n* = 7)		
Male sex, no. (%)			7	(50.0)	5	(71.4)	0.64
Age, mean (SD), y			77.6	(2.5)	79.1	(3.1)	0.24
BMI, mean (SD)			24.3	(3.8)	22.1	(2.1)	0.18
MMSE score, mean (SD)			26.7	(2.5)	26.9	(2.5)	0.9
CCI score, mean (SD)			0.6	(0.5)	0.9	(1.1)	0.41
Comorbidity besides CCI: ≥1, no. (%)			8	(57.1)	4	(57.1)	1
Cancer site, no. (%)							
Oesophagus			2	(14.3)	1	(14.3)	
Colon			1	(7.1)	1	(14.3)	
Head and neck			2	(14.3)	2	(28.6)	
Hepatobiliary and pancreatic			6	(42.9)	3	(42.9)	
Bone and soft tissue			3	(21.4)	0		
Education duration: longer than 12 years, no. (%)			5	(35.7)	7	(100.0)	<0.01
ASA class: class ≥3, no. (%)			3	(21.4)	3	(42.9)	0.35
CAGE questionnaire: ≥2, no. (%)			1	(7.1)	0		1
PS (ECOG): ≥2, no. (%)			0		0		1

ASA, American Society of Anaesthesiologists; BMI, body mass index; CAGE questionnaire, Cut, Annoyed, Guilty, Eye-opener, a screening test for potential alcohol problems; CCI, Charlson’s Comorbidity Index; MMSE, Mini-Mental State Examination; PS (ECOG), Eastern Cooperative Oncology Group performance status score; SD, standard deviation.

**Table 2:** Preventive effect of TJ-54 on the manifestation of postoperative delirium

	All ages		All ages	
Variables	Any delirium (+)/(−)	OR (95% CI)	Delirium with agitation (+)/(−)	OR (95% CI)
Ref. Placebo	21/51		10/62	
TJ54	25/52	1.17 (0.58–2.34)	10/67	0.92 (0.36–2.37)
	≥65 years		≥65 years	
Variables	Any delirium (+)/(−)	OR (95% CI)	Delirium with agitation (+)/(−)	OR (95% CI)
Ref. Placebo	17/19		9/29	
TJ54	19/24	0.94 (0.39–2.25)	8/36	0.72 (0.25–2.09)
	≥75 years		≥75 years	
Variables	Any delirium (+)/(−)	OR (95% CI)	Delirium with agitation (+)/(−)	OR (95% CI)
Ref. Placebo	5/2		5/2	
TJ54	7/7	0.40 (0.06–2.80)	3/11	0.11 (0.01–0.87)

CI, confidence interval; any delirium, delirium according to the Diagnostic and Statistical Manual of Mental Disorders 5 (DSM-5); delirium with agitation, delirium according to the DSM-5 and a score of ≥1 on the agitation subscale of the DRS-R-98; OR, odds ratio.

**Figure 1:**
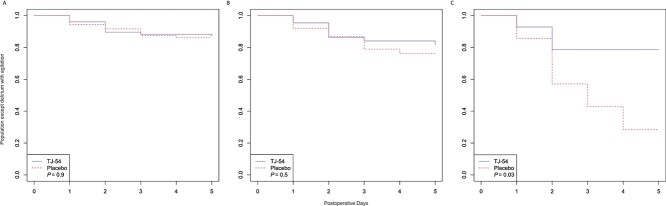
Time-to-event analysis of the effect of yokukansan (TJ-54) on the manifestation of postoperative delirium with agitation compared with that of placebo. **A**. Across all ages, there was no difference between the TJ-54 and placebo groups. **B**. In the 65 years or older group, there was no difference between the TJ-54 and placebo groups. **C**. In the 75 years or older group, the TJ-54 group showed a longer period of agitation-free delirium than that of the placebo group.

Although no significant differences in time to onset were found in the 65 years or older age group or across all ages for delirium with agitation or any delirium (Fig. 1A and B and Supplementary Figure S1), time to postoperative delirium with agitation onset was shorter in the placebo group than in the TJ-54 group in patients aged 75 years or older (*P* = 0.03, Fig. 1C).

## Discussion

This is the first study to show that yokukansan prevents the onset of postoperative delirium with agitation following highly invasive cancer resection in patients aged 75 years or older. The older adult population aged 75 years or older will account for 20% of Japan’s population in 2030 (27) and is vulnerable to surgical insults. This group commonly develops postoperative delirium and also has a high risk of postoperative cognitive decline, which is strongly associated with delirium (28). Moreover, highly invasive surgery is a major risk factor for postoperative delirium (29,30). Therefore, delirium prevention using yokukansan, a relatively safe herbal medicine (12,31), may benefit a large number of older adult patients with high delirium risk who undergo highly invasive cancer resection by decreasing delirium-related undesirable outcomes, such as cognitive decline. The alleviating effect of yokukansan on agitative behaviour is consistent with previous studies, which reported a specific feature of yokukansan in which it does not impact general symptoms (16,20). It has been suggested that yokukansan targets agitation by activating the serotonin (5HT)-1A receptor and inhibiting the 5HT-2A receptor and glutamatergic system (20). Furthermore, an acetylcholine-releasing effect of yokukansan has been reported (32). Given the probable delirium aetiology, in which benzodiazepine-derived activation of the glutamatergic system and the anticholinergic effect of medication are pivotal mechanisms underlying the cause of delirium (33), yokukansan likely modulates these pathways to induce a beneficial effect on agitation. These multifunctional pharmacological actions are derived from the ingredients and components of yokukansan (32). In particular, Geissoschizine methyl ether of Uncariae Uncis cum Ramaulau is believed to contribute to agitation management (20) and has also been speculated to benefit delirium with agitation. In addition to these known pharmacological effects of yokukansan against agitation, yokukansan has hypothetically beneficial profiles to alleviate delirium via neuroprotective functions, such as anti-inflammatory and anti-oxidant effects (32), which are also relevant to delirium pathophysiology (33). Delirium with agitation that causes psychomotor activation results in violent behaviour and other incidents that burden nursing care, such as self-extubation and falls (34). Thus, preventing delirium with agitation using yokukansan could reduce workforce burdens and promote medication adherence, which will lead to improvements in surgical outcomes.

However, when we included younger patients aged under 75 years, there were no significant differences in efficacy between yokukansan and placebo for delirium or delirium with agitation. The interaction between age and yokukansan efficacy for delirium prevention was significant, and efficacy was higher in the population aged 75 years than that in younger patients. These results suggest an age-dependent effect of yokukansan as reported previously *in vivo* (35). Furthermore, a previous clinical study (36) targeting children with pervasive developmental disorder showed significant efficacy of yokukansan in alleviating agitation; thus, low brain reserve (37) may be a consistent factor for susceptibility to the effects of yokukansan.

A strength of this secondary analysis of a randomized, double-blind, placebo-controlled study is the specific investigation of whether yokukansan prevents postoperative delirium with agitation. Because of the multifactorial nature of delirium (15), background confounding variables can have a significant effect on study results, which makes interpretation difficult. However, we performed a per-protocol analysis of a population that completed the protocol treatment and outcome evaluations in a double-blinded randomized controlled trial. Thus, background confounding variables were generally adjusted by randomization. In terms of patient characteristics, the present study showed that the yokukansan group had shorter education duration, which could have limited the effectiveness of yokukansan to prevent delirium (38); thus, the preventive effect of yokukansan observed in the current study is robust. However, our study has several limitations. Our hypothesis was tested in a small sample of patients aged 75 years and older among whom twice as many received the study drug compared with the placebo due to random allocation without adjusting for age, and the study was performed in a single cancer centre in Japan; these factors limit the generalizability of our results. In addition, yokukansan was initiated 4–8 days before surgery in the present study; therefore, the efficacy of a different protocol, such as starting medication only 1 day before surgery as is preferred for delirium prevention (39,40), is yet to be determined. However, a previous study (20) showed time-dependent efficacy of yokukansan for hostility and excitement, which indicates that a specific period of premedication may be required. Finally, the present study focused solely on the surgical insults of anaesthesia and trauma during highly invasive cancer resection. Therefore, our findings must be interpreted with caution when considering yokukansan for other types of surgery, especially cardiovascular surgery, after which delirium is believed to occur mainly because of oxidative stress (41). To further develop yokukansan as a delirium prevention strategy, a new randomized controlled trial is recommended to test whether yokukansan prevents delirium with agitation in the older adult population undergoing cancer resection within a shorter period than 6 hours, which is a common but less invasive surgery that has a lower risk of developing delirium.

In the present study, we found that yokukansan prevented the onset of postoperative delirium with agitation in an older adult population. Given the growing older adult society in Japan and worldwide, yokukansan may be valuable for optimizing postoperative recovery by reducing the incidence of delirium with agitation, alleviating burdens on the workforce and careers, and improving surgical outcomes.

## Supplementary Material

Supplementary_Figure_hyac123

Supplementary_Tables_hyac123
