# Supplementary figures and images for: Prevention of delirium with agitation by yokukansan in older adults after cancer surgery
Source: Jpn J Clin Oncol. 2022 Jul 30;52(11):1276–81. doi: 10.1093/jjco/hyac123 (PMC9631458; doi:10.1093/jjco/hyac123)

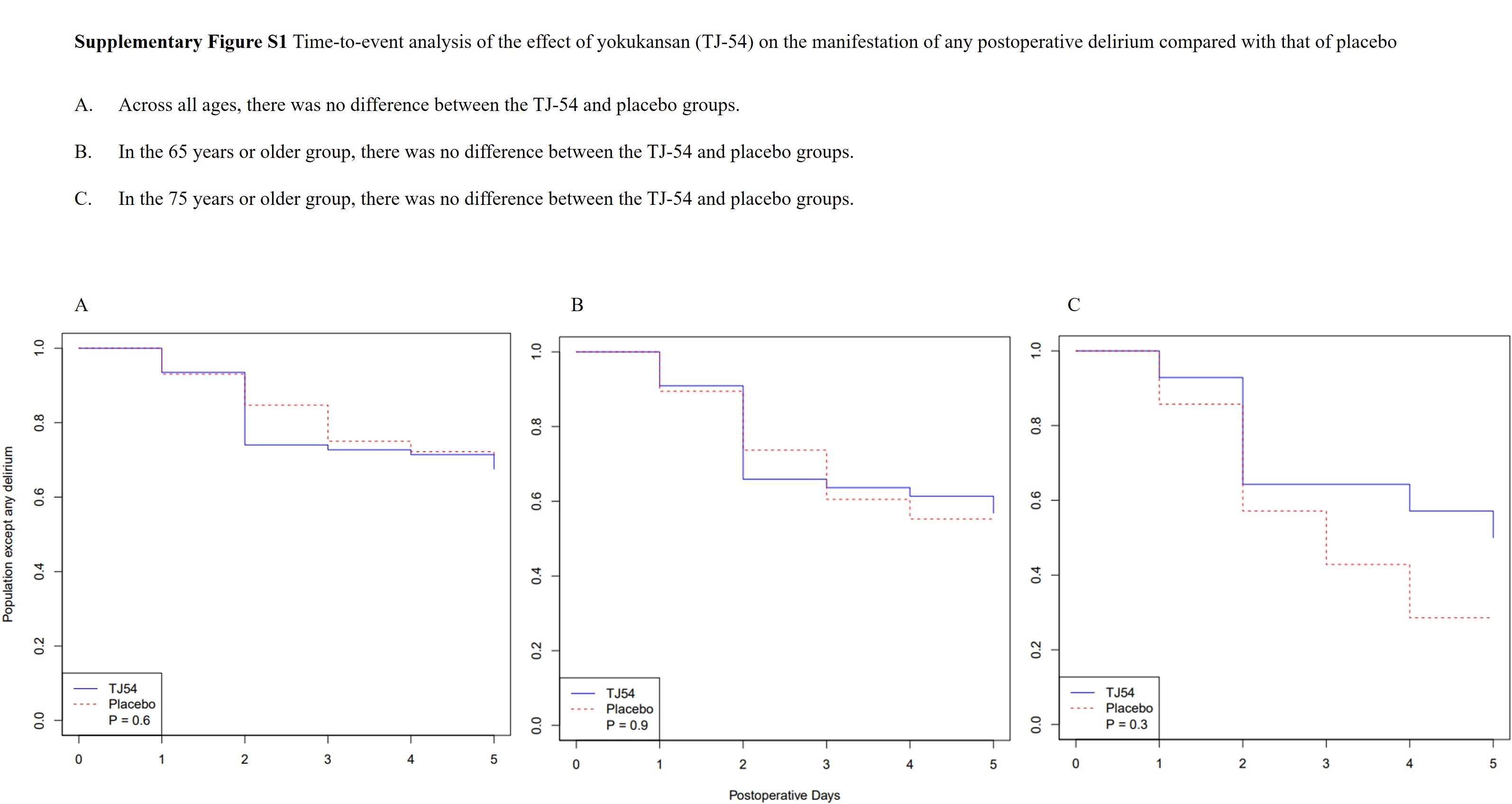

Supplement: Supplementary_Figure_hyac123 [file supplementary_figure_hyac123.jpeg]
