# Supplementary material for: Prevention of delirium with agitation by yokukansan in older adults after cancer surgery
Source: Jpn J Clin Oncol. 2022 Jul 30;52(11):1276–81. doi: 10.1093/jjco/hyac123 (PMC9631458; doi:10.1093/jjco/hyac123)
Supplement: Supplementary_Tables_hyac123 [file supplementary_tables_hyac123.doc]

**Supplementary Table 1** Characteristics of all participants

|  | | Intervention group | | Placebo group | | P value |
| --- | --- | --- | --- | --- | --- | --- |
| Characteristic | | (n = 77) | | (n = 72) | |
| Male sex, No. (%) | | 45 | (58.4) | 45 | (62.5) | 0.62 |
| Age, mean (SD), y | | 63.8 | (12.1) | 61.2 | (13.6) | 0.23 |
| BMI, mean (SD) | | 23.2 | (3.3) | 21.9 | (2.9) | 0.01 |
| MMSE score, mean (SD) | | 27.5 | (2.3) | 28.0 | (2.1) | 0.16 |
| CCI score, mean　(SD) | | 0.3 | (0.5) | 0.3 | (0.6) | 0.95 |
| Comorbidity besides CCI, No. (%) | |  |  |  |  |  |
|  | ≥1 | 22 | (28.6) | 17 | (23.6) | 0.58 |
| Cancer site, No. (%) | |  |  |  |  |  |
|  | Esophagus | 10 | (13.0) | 12 | (16.7) |  |
|  | Colon | 10 | (13.0) | 13 | (18.1) |  |
|  | Head and neck | 9 | (11.7) | 13 | (18.1) |  |
|  | Hepatobiliary and pancreatic | 26 | (33.8) | 23 | (31.9) |  |
|  | Urinary organs | 2 | (2.6) | 0 |  |  |
|  | Bone and soft tissue | 13 | (16.9) | 3 | (4.2) |  |
|  | Gynecologic | 2 | (2.6) | 7 | (9.7) |  |
|  | Other | 5 | (6.5) | 1 | (1.4) |  |
| Education background, No. (%) | |  |  |  |  |  |
|  | Longer than 12 years | 42 | (54.5) | 49 | (68.1) | 0.1 |
| ASA class, No. (%) | |  |  |  |  |  |
|  | class ≥3 | 9 | (11.7) | 13 | (18.1) | 0.36 |
| CAGE questionnaire, No. (%) | |  |  |  |  |  |
|  | ≥2 | 11 | (14.3) | 13 | (18.1) | 0.66 |
| PS (ECOG), No. (%) | |  |  |  |  |  |
|  | ≥2 | 0 |  | 0 |  |  |
| ASA, American Society of Anesthesiologists; BMI, body mass index; CAGE questionnaire, Cutting Down, Annoyed by Criticism, Guilty Feeling, Eye-openers, a screening test for potential alcohol problems; CCI, Charlson's Comorbidity Index; MMSE, Mini-Mental State Examination; PS (ECOG), Eastern Cooperative Oncology Group Performance Status Score; SD, standard deviation. | | | | | | |

**Supplementary Table 2** Characteristics of participants aged 65 years or older

|  | | Intervention group | | Placebo group | | P value |
| --- | --- | --- | --- | --- | --- | --- |
| Characteristic | | (n = 44) | | (n = 38) | |
| Male sex, No. (%) | | 26 | (59.1) | 25 | (65.8) | 0.65 |
| Age, mean (SD), y | | 72.6 | (4.5) | 71.4 | (4.8) | 0.24 |
| BMI, mean (SD) | | 22.9 | (3.2) | 21.3 | (3.2) | 0.03 |
| MMSE score, mean (SD) | | 26.7 | (2.4) | 27.6 | (2.3) | 0.08 |
| CCI score, mean　(SD) | | 0.4 | (0.5) | 0.5 | (0.7) | 0.51 |
| Comorbidity besides CCI, No. (%) | |  |  |  |  |  |
|  | ≥1 | 18 | (40.9) | 15 | (39.5) | 1.00 |
| Cancer site, No. (%) | | | | | | |
|  | Esophagus | 7 | (15.9) | 6 | (15.8) |  |
|  | Colon | 4 | (9.1) | 6 | (15.8) |  |
|  | Head and neck | 4 | (9.1) | 10 | (26.3) |  |
|  | Hepatobiliary and pancreatic | 20 | (45.5) | 14 | (36.8) |  |
|  | Urinary organs | 2 | (4.5) | 0 |  |  |
|  | Bone and soft tissue | 5 | (11.4) | 0 |  |  |
|  | Gynecologic | 0 |  | 2 | (5.3) |  |
|  | Other | 2 | (4.5) | 0 |  |  |
| Education background, No. (%) | | | | | | |
|  | Longer than 12 years | 21 | (47.7) | 25 | (65.8) | 0.12 |
| ASA class, No. (%) | | | | | | |
|  | class ≥3 | 8 | (18.2) | 11 | (28.9) | 0.30 |
| CAGE questionnaire, No. (%) | | | | | | |
|  | ≥2 | 6 | (13.6) | 3 | (7.9) | 0.49 |
| PS (ECOG), No. (%) | | | | | | |
|  | ≥2 | 0 |  | 0 |  |  |
| ASA, American Society of Anesthesiologists; BMI, body mass index; CAGE questionnaire, Cutting Down, Annoyed by Criticism, Guilty Feeling, Eye-openers, a screening test for potential alcohol problems; CCI, Charlson's Comorbidity Index; MMSE, Mini-Mental State Examination; PS (ECOG), Eastern Cooperative Oncology Group Performance Status Score; SD, standard deviation. | | | | | | |
